# Smoking cessation after hospitalization for myocardial infarction or cardiac surgery: Assessing patient interest, confidence, and physician prescribing practices

**DOI:** 10.1002/clc.23272

**Published:** 2019-10-24

**Authors:** Hayden Riley, Nitesh Ainani, Ahmad Turk, Samuel Headley, Heidi Szalai, Mihaela Stefan, Peter K. Lindenauer, Quinn R. Pack

**Affiliations:** ^1^ Division of Cardiovascular Medicine Baystate Medical Center Springfield Massachusetts; ^2^ Department of Exercise Science and Sports Studies Springfield College Springfield Massachusetts; ^3^ Cardiac and Pulmonary Rehabilitation The Miriam Hospital Providence Rhode Island; ^4^ Division of Cardiology Baystate Medical Center Springfield Massachusetts; ^5^ Institute for Health Care Delivery and Population Science Baystate Medical Center Springfield Massachusetts; ^6^ Department of Internal Medicine Baystate Medical Center Springfield Massachusetts; ^7^ University of Massachusetts Medical School at Baystate Springfield Massachusetts

## Abstract

**Background:**

Prioritizing and managing multiple behavior changes following a cardiac hospitalization can be difficult, particularly among smokers who must also overcome a serious addiction.

**Hypothesis:**

Hospitalized smokers will report a strong interest in smoking cessation (SC) but will receive little assistance from their physicians.

**Methods:**

We asked current smokers hospitalized for an acute cardiac event to prioritize their health behavior priorities, and inquired about their attitude toward SC therapies. We also assessed SC cessation prescriptions provided by their physicians.

**Results:**

Of the 105 patients approached, 81 (77%) completed the survey. Of these, 72.5% ranked SC as their greatest health change priority, surpassing all other behavior changes, including: taking medications, attending cardiac rehabilitation (CR), dieting, losing weight, and attending doctor appointments. Patients felt that SCM (44%), CR (41%), and starting exercise (35%) would increase their likelihood for SC. While most patients agreed that smoking was harmful, 16% strongly disagreed that smoking was related to their hospitalization. At discharge, medication was prescribed to ~32% of patients, with equal frequency among patients who reported interest and those who reported no interest in using medications.

**Conclusion:**

The majority of hospitalized smokers with cardiac disease want to quit smoking, desire help in doing so, and overwhelmingly rate cessation as their highest health behavior priority, although some believe smoking is unrelated to their disease. The period following an acute cardiac event appears to be a time of great receptivity to SC interventions; however, rates of providing tailored, evidence‐based interventions are disappointingly low.

## 1 INTRODUCTION

Smoking cessation (SC) provides substantial benefit to patients who are current smokers at the time of their admission in preventing subsequent events.^1^ However, patients hospitalized for a cardiac event are often advised to make multiple behavior modifications to achieve a healthier lifestyle. Research from more than 10 years ago suggest that smokers are interested in cessation; unfortunately, long‐term SC success rates are disappointingly low as more than 60% of smokers hospitalized for an acute cardiac condition continue smoking post‐discharge.^1^, ^2^, ^3^ One reason for this may be that smokers feel overwhelmed by the number of behavior changes they are recommended to make and place smoking as a secondary priority compared to other changes such as losing weight or attending cardiac rehabilitation.

Additionally, physicians and cardiologists lack training in SC and typically offer only limited support and assistance to patients to make SC a reality,^1^, ^4^, ^5^, ^6^ even though research indicates that as if patients are provided with intensive SC counseling and medications during hospitalization up to 50% of patients will be nonsmokers at 12 months.^7^ Due to these gaps, we sought to assess smokers' opinions of relative health priorities and their understanding of the relationship between smoking and cardiovascular disease. In addition, we identified which methods for SC, if any, patients would be most interested in and if they were prescribed any medications during hospitalization and at the time of hospital‐discharge. We hypothesized that hospitalized patients would be aware of the role smoking plays in the development of heart disease and would place high priority on SC at the time of their event.

## 2 METHODS

We surveyed English‐speaking patients admitted to Baystate Medical Center, a 700‐bed hospital located in Springfield, MA, who were hospitalized for acute cardiac care between October 2015 and May 2016 and were current smokers at the time of their hospital admission. A current smoker was defined as a patient reporting having had a single cigarette within the 30 days prior to hospital admission, although most use was much higher than this. Eligibility for the survey was defined as patients hospitalized with a diagnosis of myocardial infarction (MI) or who underwent a percutaneous coronary intervention (PCI), coronary artery bypass graft (CABG), with or without valve repair or replacement during their hospitalization. Patients admitted solely for heart failure were excluded.

During hospitalization, patients were asked to complete a questionnaire that was developed specifically for the purpose of this study by the two primary authors (Hayden Riley and Quinn R. Pack). The questionnaire consisted of 29 items. All questions were closely reviewed for clarity, validity, and content by co‐authors prior to being cognitively tested with eight smokers to ensure ease of use and comprehensiveness.^8^ The primary purpose of the questionnaire was to better understand patient opinion about tobacco, thus, interest in SC interventions, counseling, and/or medication prescriptions was not a requirement to participate in the survey. Patients received counseling, SC medication, or other referrals for support per usual care routines in our hospital.

Full methods of the survey, its administration, and full survey questions can be found in prior publications.^9^, ^10^ In this study, we present the results of questions 5‐6, 8‐9, and 22‐23. We believe that responses to these questions would be directly applicable to cardiologists and cardiothoracic surgeons to help them provide a tailored SC intervention to their hospitalized smokers. Questions 5, 6, and 8 used Likert Scales to assess patient interest in quitting smoking, confidence in their ability to quit, and how strongly they feel their smoking habit was related to their hospitalization, respectively. Likert scale questions were averaged, but were also reported grouped in low (1, 2), moderate (3), or high (4, 5) categories. Question 9 provided a list of SC methods (ie, medications, exercise, group support, etc.) and asked patients to select which methods, if any, they felt would help them to quit smoking. Questions 22 and 23 were used to assess health behavior priorities. In question 22, patients were asked to select which behavior change(s) they felt they should make following discharge to improve their health. A full list of the question options provided can be found in Table 1. In question 23, patients were asked to rank their selected behaviors based upon their perceived personal level of importance. A “1” was instructed to be placed next to the most important behavior selected, a “2” next to the second most important, and finally, a “3” next to the third most important.

**Table 1:** Top health priorities for smokers with an acute heart condition

Making multiple health behavior changes at the same time can often be difficult. Which of the following would you consider important behavior change(s) you should make?		Please rank your answers according to how important you think they are^b^			
	Overall^a^	First highest priority	Second highest priority	Third highest priority	Total points^c^ (max. 240)
Number of respondents	81	80	79	78	‐
Quit smoking	75 (93%)	58 (72.5%)	6 (7.6%)	8 (10.3%)	194
Consistently take prescribed medications	64 (79%)	11 (13.8%)	17 (21.5%)	12 (15.4%)	79
Attend cardiac rehab	55 (68%)	3 (3.8%)	17 (21.5%)	14 (17.9%)	57
Follow a healthy diet	61 (75%)	1 (1.3%)	19 (24.1%)	13 (16.7%)	54
Attend all doctor's appointments	62 (77%)	4 (5.0%)	6 (7.6%)	11 (14.1%)	35
Lose weight	41 (51%)	2 (2.5%)	9 (11.4%)	5 (6.4%)	29
Reduce stress	59 (73%)	1 (1.3%)	4 (5.1%)	7 (9.0%)	18
Start an exercise program	42 (52%)	0 (0.0%)	0 (0.0%)	6 (7.7%)	6
Other	5 (6%)	0 (0.0%)	1 (1.3%)	2 (2.6%)	4

a Multiple selections were possible.

b Only one selection was possible for each rank.

c Each first highest priority counted as three points, second as two points, and third as one point. These were then multiplied by the number of respondents in each category and summed. For example, quitting smoking had 58 first priority respondents, 6 second priority respondents and 8 third priority respondents such that quitting smoking had 174 pts (3*58) + 12 (2*6) + 8(1*8) for a total of 194 points out of 240 maximum points.

Patients also completed the Fagerström Test for Nicotine Dependence (FTND).^11^ Scores were calculated for the FTND based upon the tests individual scoring instructions. The FTND consists of six questions derived from the Fagerström Tolerance Questionnaire and is a self‐report measure of nicotine dependence. An FTND score of 5 to 7 indicates moderate dependence; whereas a score ≥8 indicates high nicotine dependence. Lastly, each patient's medical record was examined to determine whether patients were prescribed nicotine replacement therapy (NRT) and/or another smoking cessation medication (SCM), such as Varenicline or Bupropion, during hospitalization and at discharge. This study was approved by the Baystate Medical Center and the Springfield College institutional review boards.

## 3 STATISTICAL ANALYSIS

We used REDCap, a web‐based database, to enter, monitor, and export data.^12^ Likert scale questions were averaged but were also reported as proportions in low (1, 2), moderate (3), or high (4, 5) categories. Chi square and odds ratio analyses were used to assess the relationships between questionnaire responses, NRT/SCM prescription inpatient, and prescription at discharge. In question 23, each first highest priority counted as three points, second as two points, and third as one point. These were then multiplied by the number of respondents in each category and summed for comparison across health change categories. The FTND was scored based upon its individual scoring instructions. We used IBM SPSS version 21 and (SAS institute, Cary, North Carolina) JMP 12.0.1 for all statistical analyses. An alpha level of 0.05 was used to determine statistical significance.

## 4 RESULTS

Of the 105 patients approached, 83 (79%) consented to participate and 81 (77%) completed the questionnaires. As a group, patients were 57 years old (±10 years), 72% Caucasian, and 69% male. Patients were diagnosed with myocardial infarction and/or percutaneous coronary intervention (77%), coronary artery bypass graft and/or valve surgery (22%), and angina (1%). Risk factors were common: 19 (24%) had diabetes mellitus, 46 (57%) had hypertension, 17 (21%) had hyperlipidemia, 31 (38%) had a history of depression, and 39 (48%) had a family history of coronary artery disease. On average, patients smoked 16 ± 9 cigarettes per day for 37 ± 13 years and exhibited moderate to high levels of nicotine dependence (FTND 5.0 ± 2.4).^3^

Smokers consistently ranked SC (72.5%) as the most important behavior change they felt they should make following hospital discharge, beyond medication adherence (13.8%), cardiac rehabilitation participation (3.8%), attending all doctor's appointments (5%), etc., with little variation among the group (Table 1). In addition, most patients, 54 (67%), were confident in their ability to successfully quit and if offered, 81% of patients felt that at least one kind of cessation aid would help them to quit smoking. SC aids that patients identified as potentially helpful are found in Table 2. The most frequently reported items were SC medications, cardiac rehab, and an exercise program.

**Table 2:** Patient‐reported aids to assist smoking cessation

Which of the following, if any, do you think would help you quit smoking?	n = 81 (%)
Medications to help you quit smoking	36 (44)
Cardiac rehabilitation	33 (41)
An exercise program	28 (35)
Self‐help materials/instructions	23 (28)
Individual smoking cessation counseling	17 (21)
Education on the health effects of smoking and smoking cessation	16 (20)
None of the above	15 (19)
Other	14 (17)
Physician smoking cessation counseling	13 (16)
Group smoking cessation counseling	13 (16)
The 1‐800‐QUIT‐NOW national smoking quit line	8 (10)

*Note*: Multiple selections were possible.

Although 36 (44%) of patients reported interest in cessation medications and 30 (37%) of patients were provided at least one medication during hospitalization, we found no relationship between patient interest and physician prescription (OR 0.75 [95% CI 0.30 to 1.9], *P* = 0.53). Specifically, only 12 of the 36 (33%) patients interested in medications were prescribed NRT/SCM during their hospitalization. At the same time, 18 patients (30%) who did not express interest in medications were still prescribed NRT and/or a SCM. Inpatient medication prescription was highly predictive of medication prescription at discharge (*χ* ^2^ = 76.8, *P* < 0.001), with 1 additional patient being prescribed SCM at discharge, and no change in the other 30 patients.

Despite the strong general interest in quitting smoking among this population, 20% of patients were not interested at all in quitting smoking (Likert scale score: 1‐2) or indifferent (Likert scale score: 3). Similarly, 19% of all patients surveyed reported that they did not feel that any form of assistance would help them to quit. Lastly, 16% of patients disagreed (or strongly disagreed) that their current heart problem (the reason they were hospitalized) was related to their smoking habit, with an additional 24% reporting that they did not know whether or not there was a relationship (Table 3).

**Table 3:** Perceived impact of cigarette smoking on health

On a scale of 1 to 5 please indicate how much you agree or disagree with the following statement	N = 80	Strongly disagree (1 or 2)	Unknown (3)	Strongly agree (4 or 5)
Quitting smoking is important to my health.		2 (2.5%)	4 (5%)	74 (92.5%)
My smoking habit is related to my current health problem (the main reason I was hospitalized).		13 (16%)	19 (24%)	48 (60%)

*Note*: Answers range from 1 to 5, using the following scale: (1 = strong disagree, 5 = strongly agree).

## 5 DISCUSSION

Among patients hospitalized for a cardiac event or surgery, the overwhelming majority wanted to quit smoking, prioritized SC over other significant behavior changes following discharge, and indicated interest in a wide variety of interventions they felt would help them quit. Despite the interest patients showed in quitting smoking and the need for them to do so, patterns of prescription medications for SC therapy seemed haphazard with no relationship to patient‐reported interest. Thus, there appears to be a disconnect between patient interest in SC, and the provision of appropriate resources they want and need, including pharmacotherapy, to aid in a successful cessation attempt. Given that not every smoker was interested in every therapy option, our results also suggest that a tailored approach to SC is imperative to meet the needs of our patients.

Patients hospitalized for an acute cardiac event are often faced with multiple health behavior changes and numerous recommendations following discharge, which may be difficult to manage simultaneously. Previous research has shown that there is a general reluctance to address multiple risk factors, even when several are present.^13^ To our knowledge, this is the first study to evaluate health behavior priorities among current smokers trying to manage these competing demands. Our study showed that smokers overwhelmingly chose SC as their highest priority following discharge, which may indicate that patients are inherently aware of the importance of quitting smoking in regard to their health. In addition, we believe this is also the first to survey of patients hospitalized specifically with cardiac disease. However, our findings are consistent with previous literature among the general (noncardiac) population, which shows that a large majority of smokers are interested in quitting smoking and motivated to do so during hospitalization.^14^, ^15^, ^16^, ^17^, ^18^ This may be because smokers may be more motivated to quit during hospitalization due to the increase in personal relevance (ie, sentinel event) their illness or symptoms provide.^17^

Unfortunately, despite patients' interest in quitting and the fact that SC is the single most effective lifestyle change a patient can make after myocardial infarction, up to 60% of patients who smoke will eventually relapse within 1 year of hospitalization, most of whom will do so within the first 3 weeks following discharge.^1^, ^14^, ^15^, ^16^, ^17^, ^19^ These two facts only reinforce the notion that starting treatment during hospitalization is critical. Furthermore, SC support, such as SCM and NRT, are readily available throughout a patient's hospital stay.^18^ However, a single in‐hospital intervention is unlikely to be effective. Rather, meta‐analyses show that the most successful interventions for supporting long‐term abstinence are initiated during hospitalization and then continued for at least 1 month following discharge,^20^ in programs such as cardiac rehabilitation.

Based upon the results of this study, we believe that SC intervention should start with an assessment of the patient's understanding and education, as well as their intervention interests. Considering that a portion of our patient population (16%) did not believe their heart disease was caused in part by smoking, and an additional 24% stated that they were unsure if there was a relationship between the two, it seems that in‐hospital treatment should incorporate the education necessary to eliminate any misunderstanding. Additionally, some patients indicated that they felt nothing would help them to quit smoking. Previous research has shown that two‐thirds of smokers often attempt to quit without seeking treatment or assistance, despite the increase in success cessation aids provide.^21^ One study found that SC counseling provided by a physician trained in motivational interviewing increased uptake of SC counseling, exposure to NRT, and a significant increase in smoking abstinence at 12 months.^7^ Thus, it appears imperative and beneficial that physicians counsel their patients on SC, and use a variety of aids to achieve this outcome.

Considering the multitude of resources available to improve SC outcomes, we asked patients in our study what they felt would help them quit smoking. Patients felt that cessation medications would be the most useful. However, our study also showed that there is currently a large treatment gap in the prescription of SCM and NRT provided to hospitalized smokers, even if they indicate interest in its use. For instance, previous literature indicates that when initiated in‐hospital following acute coronary syndrome, Varenicline significantly increases SC 17, 22; however, in the present study, varenicline was only prescribed to one (1.2%) patient. Overall, in our study, only about one‐third (37%) of the patients were prescribed NRT or SCM while inpatient or at discharge. This compares favorably (but is still unacceptably low) to another larger study in which only 22.7% of smokers (n = 366 675) among 285 US hospitals received one SCM during hospitalization. Considering the fact that the Affordable Care Act mandated that tobacco treatment be provided at no additional cost under most health insurance plans,^23^ it appears that physicians are missing opportunities to provide an effective and affordable option for SC treatment. Furthermore, with e‐Cigarette use on the rise,^24^ patients may be enticed to use these devices as a SC tool. Information regarding the use of these devices as a SC aid are inconsistent; however, one recent study found that e‐cigarettes were more effective for SC when compared to NRT,^25^ but that 80% of patients in the e‐cigarette group were still vaping a year later. Even though e‐cigarette use appears to carry fewer potential risks compared to traditional cigarettes, long‐term health effects remain unknown, thus making continued long‐term use potentially problematic.^26^ Considering the uncertainty surrounding the efficacy and safety of e‐cigarettes, FDA approved SCM, such as NRT, Varenicline, and Bupropion, should be encouraged as first‐line pharmacotherapy treatment options.^27^

Considering that SC counseling rates vary widely from 17% to 81% and pharmacotherapy administration rates during hospitalization are low, 28 it appears that physicians are not capitalizing on key opportunities to assist their patients in quitting smoking. Previous research indicates that, although providers recognize the benefits of SCM/NRT, they are reluctant to implement these treatments due to lack of knowledge and prescribing experience, concerns surrounding the financial implication of providing NRT to patients, and the possibility of liability and legal implications.^29^ However, recent literature indicates that lingering safety concerns surrounding the use of SCM and NRT no longer appear justified.^30^, ^31^ Additionally, failure to provide SC counseling has often been attributed to competing priorities and busy work, but this common notion is not generally supported by empirical research^32^ and appears to be more a reflection of physician priorities. Thus, we believe that engaging patients during key teachable moments, such as hospitalization, should be a priority among all clinicians in an effort to eliminate their patients most significant risk factor for heart disease. And we strongly encourage all healthcare providers to seek the training necessary to overcome any hesitancies and effectively deliver SC support in the hospital. Likewise, assessing patients and assisting in their quit attempt must be universal, rather than selective, and tailored to the patient's interests and needs.^27^

The primary limitation of this study is that our sample size was small and limited to only English‐speaking patients. In addition, this study was performed at a single center in western Massachusetts, therefore, our results may not uniformly apply to other institutions, clinical settings, and/or patient populations. An additional limitation of the study is that 22 patients refused to participate. Even though our participation rate was high (79%), our questionnaire answers may not fully represent all smokers hospitalized with an acute cardiac condition. In addition, information regarding other interventions performed during hospitalization, such as SC counseling, was not collected as a part of this study. Lastly, social desirability bias may be of concern because patients knew this was a study about SC and may have given more “favorable” answers to our survey questions.

## 6 CONCLUSION

All healthcare providers have the opportunity to reduce the tremendous burden smoking places on the health of their patients; however, doing so requires translating the use of NRT, SCM, and other treatment options into routine practice. Given the teachable moment that hospitalization provides, treatment would ideally be initiated at this time and coupled with at least 1 month of follow‐up care in programs such as cardiac rehabilitation. Based upon the results of our study, we hope that physicians become more confident and consistent with pharmacotherapy application among this population, otherwise, they will continue to miss a significant opportunity to improve the morbidity and mortality of their patients.

## CONFLICT OF INTEREST

The authors declare no potential conflict of interests.
